# RNA-Seq Analysis of Abdominal Fat Reveals Differences between Modern Commercial Broiler Chickens with High and Low Feed Efficiencies

**DOI:** 10.1371/journal.pone.0135810

**Published:** 2015-08-21

**Authors:** Zhu Zhuo, Susan J. Lamont, William R. Lee, Behnam Abasht

**Affiliations:** 1 Department of Animal & Food Sciences, University of Delaware, Newark, Delaware, United States of America; 2 Department of Animal Science, Iowa State University, Ames, Iowa, United States of America; 3 Maple Leaf Farms, Inc., Leesburg, Indiana, United States of America; Humboldt-University Berlin, GERMANY

## Abstract

For economic and environmental reasons, chickens with superior feed efficiency (FE) are preferred in the broiler chicken industry. High FE (HFE) chickens typically have reduced abdominal fat, the major adipose tissue in chickens. In addition to its function of energy storage, adipose tissue is a metabolically active organ that also possesses endocrine and immune regulatory functions. It plays a central role in maintaining energy homeostasis. Comprehensive understanding of the gene expression in the adipose tissue and the biological basis of FE are of significance to optimize selection and breeding strategies. Through gene expression profiling of abdominal fat from high and low FE (LFE) commercial broiler chickens, the present study aimed to characterize the differences of gene expression between HFE and LFE chickens. mRNA-seq analysis was carried out on the total RNA of abdominal fat from 10 HFE and 12 LFE commercial broiler chickens, and 1.48 billion of 75-base sequence reads were generated in total. On average, 11,565 genes were expressed (>5 reads/gene/sample) in the abdominal fat tissue, of which 286 genes were differentially expressed (DE) at q (False Discover Rate) < 0.05 and fold change > 1.3 between HFE and LFE chickens. Expression levels from RNA-seq were confirmed with the NanoString nCounter analysis system. Functional analysis showed that the DE genes were significantly (p < 0.01) enriched in lipid metabolism, coagulation, and immune regulation pathways. Specifically, the LFE chickens had higher expression of lipid synthesis genes and lower expression of triglyceride hydrolysis and cholesterol transport genes. In conclusion, our study reveals the overall differences of gene expression in the abdominal fat from HFE and LFE chickens, and the results suggest that the divergent expression of lipid metabolism genes represents the major differences.

## Background

Feed efficiency (FE)–the efficiency of converting feed intake to body weight gain–is of great importance to modern commercial broiler chicken production. Feed cost is the major expense for chicken production and represents up to 70% of the total economic input. For a unit of weight gain, HFE chickens consume less feed and produce less excrement [1]. Therefore, improving FE could reduce cost of production and waste management and lower emission of nitrogenous and greenhouse gases. A comprehensive understanding of the biological mechanisms controlling FE is crucial to develop optimal breeding and selection strategies. Previous studies on chicken FE have investigated gene expression in breast muscle by microarray [2–4] and by RNA-Seq [5], but gene expression associated with FE in adipose tissue is still unexamined.

Adipose tissue is now recognized as a metabolically active endocrine organ and plays a central role in energy homeostasis. It serves as the major site for lipid deposition and lipid metabolism. Adipose-derived hormones, proteins, and other biologically active factors regulate metabolic and immune activities locally and systemically (reviewed in [6,7]). Given that obesity and obesity-related conditions are prevalent worldwide, a thorough understanding of adipose biology is needed to prevent and intervene the disease. The chicken has been proposed as a model for adiposity studies, as it possesses unique features relevant to obesity research. Different from rodents adipose tissue, human adipose tissue has a relatively limited lipogenic capacity [8]. Similarly, chicken adipose tissue is not recognized as the major organ for *de novo* lipid synthesis [9]. The majority of lipids accumulated in adipose tissue are synthesized in the liver, circulated in the blood stream, and then absorbed by adipose tissue [10,11]. The chicken adipose tissue is insensitive to insulin [12,13], similar to the physiological behavior of adipose tissue of obese people and type 2 diabetes patients.

A few studies have examined the global gene expression of chicken adipose tissue by using microarray technology. By comparing the gene expression of fat line and lean line chickens that were divergently selected for abdominal fat content for seven generations, the gene expression related to adipogenesis and lipogenesis were found to be up-regulated in fat line chickens, but gluconeogenesis or glycolysis genes were down-regulated [14,15]. In commercial broiler chickens, fasting and insulin neutralization affected the expression of adipogenic genes and enhanced lipid oxidization in adipose tissue [16]. Genes involved in immune response were found differentially expressed in different ages of broiler chickens [17]. Compared with commercial broilers, relatively lean chicken lines, Fayoumi and Leghorn, had higher expression of lipolysis and fatty acid oxidation genes [18].

The present study aimed to investigate gene expression in the adipose tissue associated with FE. Through profiling the gene expression of abdominal fat from selected HFE and LFE chickens using RNA-seq, we identified 286 differentially expressed (DE) genes. We paid special attention to the DE genes and pathways involved in lipid metabolism and interpreted how they contributed to the differences in adiposity between LFE and HFE chickens. Overall, our study provides insights into the relationships between feed efficiency and gene expression in abdominal fat and contributes to the understanding of the gene expression in chicken adipose tissue.

## Methods and Materials

### Experimental animals and tissue collection

A live animal experiment of 2400 commercial broiler chickens was previously conducted and used for studying various aspects of quantitative traits in broiler chickens ([5] and unpublished data). The chickens were sampled from 6 commercial broiler farms (400 chickens per farm) in the Delmarva region (USA) at 29-day age. Then the chickens were transferred to an experimental station, where each chicken was kept in a separate cage for individual feed efficiency measurement and fed *ad libitum*. The cages in the experimental station were arranged in rows at two levels, i.e. top or bottom levels, relative to their distance from the floor, and each row had 100 cages. The weight of feeders and chickens were measured and recorded at the beginning (day 29) and the end (day 46) of the test. Dead (1.5%) and sickly (0.9%) chickens were removed or culled routinely during the test. At day 47, the chickens were euthanized by manual cervical dislocation for tissue sampling. About 1 g of adipose tissue was harvested and immediately frozen in liquid nitrogen, and kept at –80°C for further RNA isolation. Fat in abdominal cavity and around gizzard were dissected and weighed after keeping the carcasses at 4°C for 24 hours. The protocols were approved by the University of Delaware Agricultural Animal Care and Use Committee.

### Calculation of feed efficiency and phenotypic correlations

Before estimating FE and correlations between FE and other phenotypic measurements, inaccurate data (1.6% of the total) resulting form artifacts was excluded. In addition, the following criteria were applied to exclude outliers (2.0% of the total) in each group. First, residual weight gain was calculated by adjusting weight gain for cage location effect. Chickens with a residual weight gain that fell outside of the mean ±3 standard deviations (SDs) were excluded. Then, residual feed consumption (RFC) was estimated as a measure of FE by calculating the difference between the actual and expected feed intake using the following equation:

$$RFC=FC-(a+b1*BW_{29}+b2*BW_{46}+Level+Row(Level)),$$

where FC is the actual feed consumption; BW_29_ and BW_46_ are the body weights at 29 and 46 days of age, respectively; Level represents the fixed effect of row location (top or bottom level) and Row(Level) represents the fixed effect of row nested within row location; and a is the intercept, b1 and b2 are the partial regression coefficients of BW_29_ and BW_46_, respectively. Chickens with RFC lying outside of the mean ± 3 SDs were excluded to eliminate the data points that might affect the accuracy of estimating RFC. As a result, data from 2254 chickens remained, and new RFC of each bird was calculated using the same model.

Within each experimental group, the birds were ranked by RFC. The chickens with extreme RFC values at both ends, designated as HFE and LFE, respectively, were selected for RNA-seq. The birds with defects (wooden breast muscle [19], leg and wings problem, etc.) were excluded. In total, 12 HFE and 12 LFE chickens were chosen (S1 Table), but two HFE samples did not generate adequate cDNA libraries. Thus, only 10 HFE and 12 LFE were used for RNA-seq, but all the chosen samples (12 HFE and 12 LFE) were used for the NanoString confirmation. The correlation coefficients between FE, feed conversion ratio (FCR), body weight, weight gain, abdominal fat weight, and abdominal fat percentage, as well as the p-values for t-tests between HFE and LFE phenotypes, were estimated using JMP (Version 11.0.0.). A threshold of p-values less than 0.05 were applied to declare significance in the data analysis.

### Total RNA extraction and cDNA library preparation

The fat samples of the selected birds were ground in frozen state in liquid nitrogen. Total RNA was extracted from ~70 mg of samples with the mirVana miRNA Isolation Kit (Life Technologies). The concentration of RNA samples was measured using the NanoDrop 1000 (Thermo Scientific). Agilent Bioanalyzer 2100 (Agilent Technologies) was utilized to assess the integrity of the total RNA. The RNA integrity number (RIN) of all samples was greater than 8.

cDNA libraries were constructed using a TruSeq Stranded mRNA LT Sample Prep Kit. Briefly, mRNA was isolated from 2 μg of total RNA using poly-T oligo-attached magnetic beads and fragmented by divalent cation. The first strand cDNA was synthesized using reverse transcriptase (Life Technologies) and random primers, followed by removal of template RNA using RNase H. During the second strand synthesis, dUTPs were used in the reaction instead of dTTPs. The double-stranded cDNA was recovered using AMPure beads (Beckman Coulter). After reverse transcription, a single ‘A’ nucleotide was added to the 3' ends of the blunt fragments to prevent them from ligating to one another during adapter ligation reaction. Then, adaptors with index were ligated to the fragments, as a corresponding single ‘T’ nucleotide on the 3' end of the adapter provided a complementary overhang for ligating the adapter to the fragment. Of note, a unique indexing adaptor was used for each sample. After clean up using AMPure beads, DNA fragments with adapter sequences were enriched by PCR. dUTP prevented the second strand cDNA from elongating due to the specificity of the enzyme, leaving only the first-strand cDNA to be amplified. Finally, the concentration of cDNA libraries was measured using a NanoDrop 1000, and the quality of the cDNA libraries was further validated using an Agilent Bioanalyzer 2100.

### Sequencing strategy

The concentration of the 22 cDNA libraries was normalized to 10 nm/μl using Tris buffer (Tris-Cl 10mM, 0.1% Tween 20, pH 8.5), as suggested by the manufacturer. Ten microliter of each uniquely-indexed, normalized library was pooled into a single sample, and the resultant pool was sequenced on four lanes of a flow cell for 75 cycles with the paired-end sequencing protocol of the Illumina Hiseq 2000 system. The resultant data was deposited in NCBI’s Short Read Archive (SRA) database (Accession SRP058295).

### QC and reads alignment

First, the RNA-seq reads of each sample were discriminated (i.e. demultiplexed) based on the indexing adaptors, and then processed with FastQC v0.10.1 to check the quality of raw sequence reads [20]. The reads were mapped to the chicken reference genome Gallus_gallus-4.0 (Ensembl, database version 78.4) using TopHat v2.0.4 [21], a fast splice junction mapper for RNA-seq reads. Parameters of TopHat were set to allow only unique alignment to the reference genome. Reads with more than two mismatches were discarded, and concordant mapping for both reads in a pair was required. To obtain the mapping statistics, the alignment BAM files were further examined using RNA-SeQC v1.1.7 [22]

### Differential gene expression and functional analysis

The genes differentially expressed (DE genes) between HFE and LFE groups were identified using Cuffdiff v2.1.1 [23]. To identify over-represented pathways and networks, and to predict the activation and inhibition states of upstream regulators, the DE genes were analyzed using the Ingenuity Pathways Analysis (IPA) system [24]. Based on the FPKM values of all genes reported by Cuffdiff, a 2-way hierarchical clustering (Ward method) of samples was performed in JMP (Version 11.0.0.).

### Confirmation of RNA-Seq data by nCounter analysis system

Expression results obtained from RNA-Seq were confirmed by the NanoString’s nCounter analysis system [25]. To gain comprehensive evaluation of the RNA-seq expression data, a set of 204 genes were chosen for nCounter probe design based on multiple ongoing RNA-seq experiments in our laboratory (S2 Table). From the same RNA samples used for RNA-Seq library constructions, 300 ng of total RNA were submitted to NanoString Technologies for hybridization, detection, and scanning. For data analysis, no background subtraction was performed since the spike-in negative controls showed a low background noise. Twelve reference genes were chosen based on coefficients of variation among all genes. The raw gene counts for each transcript were normalized by External RNA Control Consortium (ERCC) spike-in positive controls and by the reference genes. Of the 204 genes chosen, 65 were identified with low number of alignments for performing statistical test in Cuffdiff analysis and thus excluded from data analysis. The other 139 genes, containing 12 designated housekeeping genes, 31 DE genes and 96 non-DE genes based on RNA-Seq, were used for correlation analysis.The Pearson correlation coefficients of log2 (fold change) between normalized gene count and FPKM were calculated in JMP (Version 11.0.0.).

## Results

### Phenotypes

In the present study, 2400 commercial broiler chickens were hatched and raised for feed efficiency tests. Weight gain (WG), abdominal fat percentage, FCR, and RFC were calculated based on the records of body weight (BW), feed consumption (FC) and abdominal fat weight. WG had a weak correlation with BW_29_ (r = 0.23) and a strong correlation with BW_46_ (r = 0.81). Similarly, FC had a moderate correlation with BW_29_ (r = 0.39) but a strong correlation with BW_46_ (r = 0.69). Further, WG and FC had a strong correlation (r = 0.85 and r^2^ = 0.72), indicating that 72% of the variability of weight gain can be explained by FC. Moreover, abdominal fat percentage had a moderate correlation with FCR (r = 0.31) and RFC (r = 0.40) (S3 Table), which is consistent with previous reports that LFE chickens have an overall more fat deposition [26–28].

Average BW_29_, BW_46_, FC, WG, breast muscle weight, abdominal fat weight, percentage of abdominal fat, and RFC of selected chickens are summarized in Table 1. The RFC of HFE and LFE groups were significantly different, which were the basis used to select chickens for RNA-seq. There were no significant differences of initial body weight (BW_29_) and final body weight (BW_46_) between HFE and LFE groups. However, FC (p < 0.001), WG (p = 0.0035), breast muscle weight (p = 0.0361), abdominal fat weight (p = 0.0012), and abdominal fat percentage (p = 0.0040) were significantly different between HFE and LFE groups. Also of important fact is that HFE and LFE chickens do not necessarily retain the lowest or highest abdominal fat percentage, which is in concert with the moderate correlation between abdominal fat percentage and RFC. In summary, on average, the LFE birds consumed more feed and deposited less breast muscle but accumulated more abdominal fat.

**Table 1:** Phenotypic data of samples used in RNA-seq (Mean ± S.E.)

	BW_46_ (kg)	FC (kg)	WG (kg)	Breast muscle percentage (%BW)	Fat percentage (%BW)	FCR	RFC (kg)
HFE	3.12±0.07	2.91±0.05*	1.81±0.04*	23.46±0.48*	1.52±0.15*	1.61±0.02*	-0.28±0.01*
LFE	3.03±0.06	3.34±0.05*	1.62±0.04*	21.75±0.44*	2.36±0.13*	2.07±0.02*	0.36±0.01*

* Indicates significant difference (t-test, p<0.01) between HFE and LFE groups. Calculations of FCR and RFC are described in Methods and Materials. Abbreviations: BW_46_: body weight at Day 46; FC: Feed consumption; WG: Weight gain; FCR: Feed conversion ration; RFC: Residual fee consumption.

### Gene expression profiles of HFE and LFE chickens

**Fig 1:**
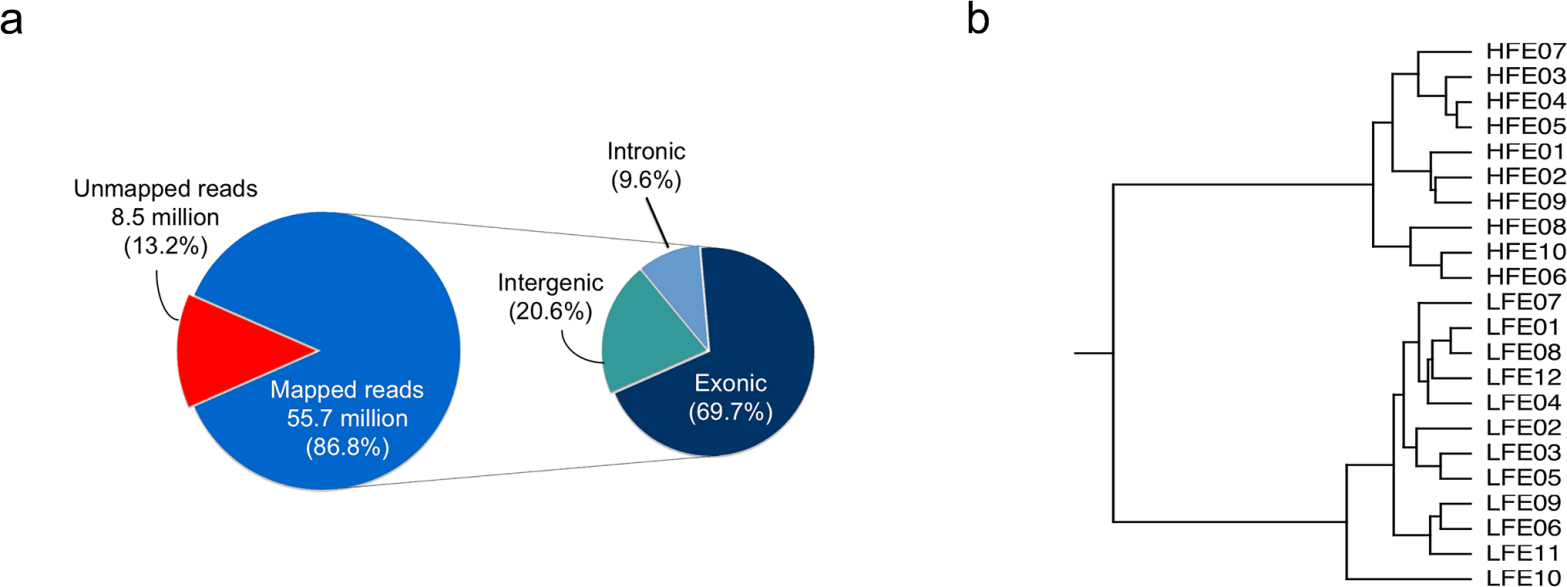
Summary of RNA-Seq data. a. Average mapping statistics. b. Hierarchical clustering of samples based on gene expression profile.

In total, 1.48 billion of 75-base sequence reads were generated, and for each sample approximately 64 million (ranging from 45 to 76 million) reads were obtained (S1 Fig). Further, 86.8% of the reads from each sample were mapped uniquely to the chicken reference genome (Ensembl Galgal4). Among the mapped reads, 60.5% of the total reads were mapped to exon regions, 17.9% were mapped to the intergenic regions, and 8.3% were mapped to intronic regions (Fig 1A). A total of 11,565 genes with at least five reads mapped per sample were detected in the RNA-seq libraries. The hierarchical clustering of samples based on the expression of all genes is presented in Fig 1B.

### Consistency of samples within groups

To avoid the expression results being affected by outlier samples, we performed a separate systematic evaluation of consistency of all samples in the HFE and LFE groups. The correlation analysis based on the gene expression profiles found that one LFE sample (#LFE10) had the lowest correlation (r = 0.85) with other samples in the LFE group, whereas the correlations among other LFE samples were about 0.94 (S4 Table). Sample LFE10 also had a lower correlation with HFE chickens when compared with other LFE samples. Consistently, hierarchical clustering results suggested that sample LFE10 was located on an isolated branch (Fig 1B). The RNA-seq data and Nanostring results showed a correlation of 0.79 for this sample (compared with an average correlation coefficient of 0.78 between RNA-seq and NanoString), confirming that the RNA-seq data for sample LFE10 was reliable. The phenotypic data didn’t rule out sample LFE10 as an outlier; however, sample LFE10 had the lowest WG and breast muscle percentage among all selected chickens. It is likely that chicken LFE10 had a certain morbidity condition with unobservable symptoms, causing the deficiency in gaining weight and building breast muscle. Therefore, sample LFE10 might be an interesting case for further studies to investigate its uniqueness of gene expression patterns and the causes, but for the purpose of the present study, it was excluded from the further DE gene analysis. As a result, a total of 10 samples from the HFE group and 11 samples from the LFE group were used for DE gene analysis.

### Identification and functional analysis of DE genes

Differential expression analysis between the HFE and LFE groups was carried out using Cuffdiff software. A total of 286 genes were found to be differentially expressed between HFE and LFE groups with a fold change larger than 1.3 at q (false discover rate) < 0.05. Of these genes, 147 were up-regulated and 139 were down-regulated in LFE group (S5 Table). The top ten up- and down-regulated genes are listed in Table 2.

**Table 2:** Top 10 up- and down-regulated genes in LFE group.

Ensembl gene ID	*Gene name*	Fold Change
Up-regulated genes		FPKM_LFE_ / FPKM_HFE_
ENSGALG00000009118	*PIT 54*	**↑**11.8
ENSGALG00000009266	*FGA*	**↑**10.6
ENSGALG00000008601	*AHSG*	**↑**10.4
ENSGALG00000003957	*APOH*	**↑**9.9
ENSGALG00000020180	*ALB*	**↑**9.8
ENSGALG00000019845	*GAL9*	**↑**9.7
ENSGALG00000008973	*AMBP*	**↑**9.2
ENSGALG00000011612	*GC*	**↑**9.0
ENSGALG00000009262	*FGB*	**↑**8.6
ENSGALG00000016667	*GAL10*	**↑**7.2
Down-regulated genes		FPKM_HFE_ / FPKM_LFE_
ENSGALG00000002614	*Unnamed*	**↓**3.4
ENSGALG00000012670	*NRSN1*	**↓**2.8
ENSGALG00000023622	*AVD*	**↓**2.6
ENSGALG00000016364	*FAM150B*	**↓**2.5
ENSGALG00000003212	*TSPO2*	**↓**2.2
ENSGALG00000019325	*Unnamed*	**↓**2.2
ENSGALG00000029151	*ISLR2*	**↓**2.1
ENSGALG00000026075	*AMER3*	**↓**2.0
ENSGALG00000001417	*CYP11A1*	**↓**2.0
ENSGALG00000015166	*GCNT1*	**↓**1.9

**↑** indicates up-regulation in LFE group

**↓** indicates down-regulation in LFE group

The DE gene list was analyzed using the IPA web application. A summary of IPA results is presented in Table 3 and Table 4. The noteworthy networks and functions identified include developmental disorder, hereditary disorder, cell-to-cell signaling and interaction, immune cell trafficking, inflammatory response, and lipid metabolism. There were 17 significant canonical pathways (p < 0.01) (The top 10 is shown in Table 5 **)**, which are involved in lipid metabolism, immune regulation, blood coagulation, and amino acid biosynthesis. The details of networks, functions, pathways, and related genes are further disused in the text.

**Table 3:** Top networks from IPA results.

ID	Associated Network Functions	Score^1^
1	Developmental Disorder, Hematological Disease, Hereditary Disorder	40
2	Cardiovascular System Development and Function, Organismal Development, Cell-to-Cell Signaling and Interaction	35
3	Drug Metabolism, Lipid Metabolism, Molecular Transport	33
4	Organismal Injury and Abnormalities, Tissue Morphology, Reproductive System Development and Function	28
5	Cellular Movement, Immune Cell Trafficking, Inflammatory Response	27

^1^Scores were calculated by IPA to rank the relevancy of DE genes and networks.

**Table 4:** Top molecular and cellular functions.

Name	p-value^1^	# molecule^2^
Lipid Metabolism	2.31E-07–6.53E-03	30
Molecular Transport	2.31E-07–6.53E-03	31
Small Molecule Biochemistry	2.31E-07–7.46E-03	34
Vitamin and Mineral Metabolism	3.37E-06–5.54E-03	12
Cellular Movement	4.52E-06–7.17E-03	31

^1^p-values were calculated with a Fisher-extract test contingency table by IPA.

^2^# molecule indicates the number of DE genes involved in the molecular and cellular function

**Table 5:** Top 10 canonical pathways.

Ingenuity canonical pathways	p-value^1^	Ratio^2^
LXR/RXR activation	1.00E-10	1.01E-01
Acute phase response signaling	5.25E-07	6.63E-02
Cholesterol biosynthesis i	1.07E-05	1.00E-01
Cholesterol biosynthesis ii (via 24,25-dihydrolanosterol)	1.07E-05	1.00E-01
Cholesterol biosynthesis iii (via desmosterol)	1.07E-05	1.00E-01
Superpathway of cholesterol biosynthesis	2.04E-05	5.75E-02
Extrinsic prothrombin activation pathway	2.63E-05	1.82E-01
Zymosterol biosynthesis	2.82E-05	1.36E-01
Intrinsic prothrombin activation pathway	2.63E-04	1.08E-01
Oleate biosynthesis ii (animals)	2.95E-04	1.67E-01

^1^p-values were calculated with a Fisher-extract test contingency table by IPA.

^2^Ratio = number of DE genes mapped to the pathway/total number of genes of the pathway.

### Higher accumulation of lipid in LFE birds

**Fig 2:**
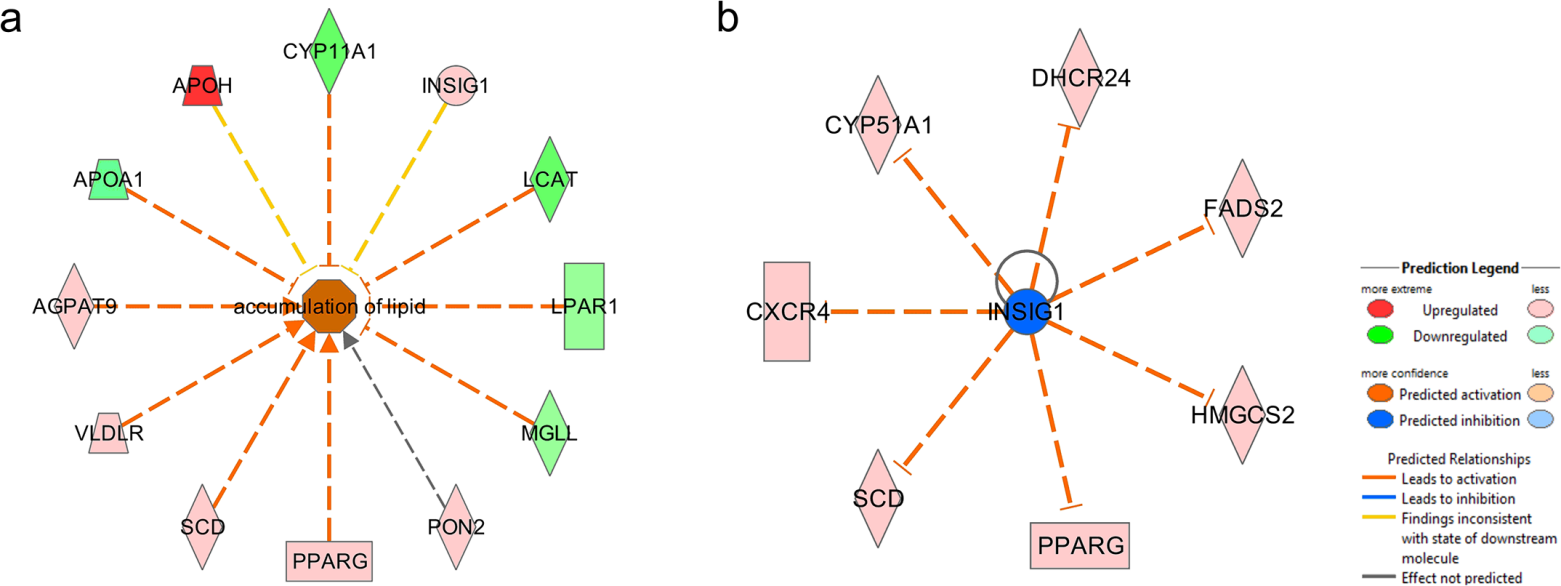
The DE genes involved in accumulation of lipid and upstream regulator INSIG1. a. The accumulation of lipid is predicted to be activated in LFE group. b. Upstream regulator INSIG1. Cholesterol biosynthesis regulator INSIG1 is predicted to be inhibited in LFE chickens.

The mean abdominal fat weight and percentage of the LFE group were significantly larger than that of the HFE group. This can be attributed to an overall higher accumulation of lipid in LFE birds (Fig 2A). Among the DE genes, a lipid hydrolysis gene [*monoglyceride lipase* (*MGLL*)] and genes involved in high-density lipoprotein (HDL) synthesis [*lecithin-cholesterol acyltransferase* (*LCAT*), *apolipoprotein A-I* (*APOA1*), and lysophosphatidic *acid receptor 1* (*LPAR1*)] and steroid hormone synthesis [*cytochrome P450*, *family 11*, *subfamily A* (*CYP11A1*)] were down-regulated, whereas lipid synthesis genes [*1*-acylglycerol-*3-phosphate O-acyltransferase 9* (*AGPAT9*), *stearoyl-CoA desaturase (delta-9-desaturase)* (*SCD*), and *diacylglycerol O-acyltransferase homolog 2 (mouse)* (*DGAT2*)] and a gene that stimulates the uptake of fatty acids and adipogenesis [peroxisome *proliferator-activated receptor gamma* (*PPARG*)] were up-regulated in LFE group. These findings suggest that the up-regulation of genes involved in lipid synthesis and the down-regulation of genes involved in triglyceride hydrolysis and reverse cholesterol transport from adipose tissue were responsible for the higher accumulation of lipid in abdominal fat in LFE group.

### Over-represented pathways

The IPA identified 17 canonical pathways that were significant with a p-value less than 0.01. These pathways are involved in lipid metabolism (LXR/RXR activation, oleate biosynthesis II), cholesterol biosynthesis (cholesterol biosynthesis I/II/III, superpathway of cholesterol biosynthesis, zymosterol biosyntheis), amino acid synthesis (serine biosynthesis, superpathway of serine and glycine biosynthesis), coagulation (intrinsic/extrinsic prothrombin activation pathway, coagulation system), and endocrine functions (estrogen biosynthesis, atherosclerosis signaling, axonal guidance signaling, retinoate biosynthesis I). These pathways will be selectively discussed further later in the text.

### Upstream regulators

Based on the DE genes, five transcription regulators [(*HNF 1 homeobox A* (*HNF1A*), *sterol regulatory element binding transcription factor 1* (*SREBF1*), *sterol regulatory element binding transcription factor 2* (*SREBF2*), *E2F transcription factor 1* (*E2F1*), and *fibroblast growth factor 2* (*FGF2*)], Tcf 1/3/4, and SREBP cleavage-activating protein (SCAP) were predicted to be activated in LFE group. The genes *phosphatase and tensin homolog* (*PTEN*), *interleukin 1* (*IL1*), *Tumor protein p53 (TP53)*, and *insulin induced gene 1* (*INSIG1*) (Fig 2B) were predicted to be inhibited in LFE group.

### Confirmation of RNA-seq experiment

**Fig 3:**
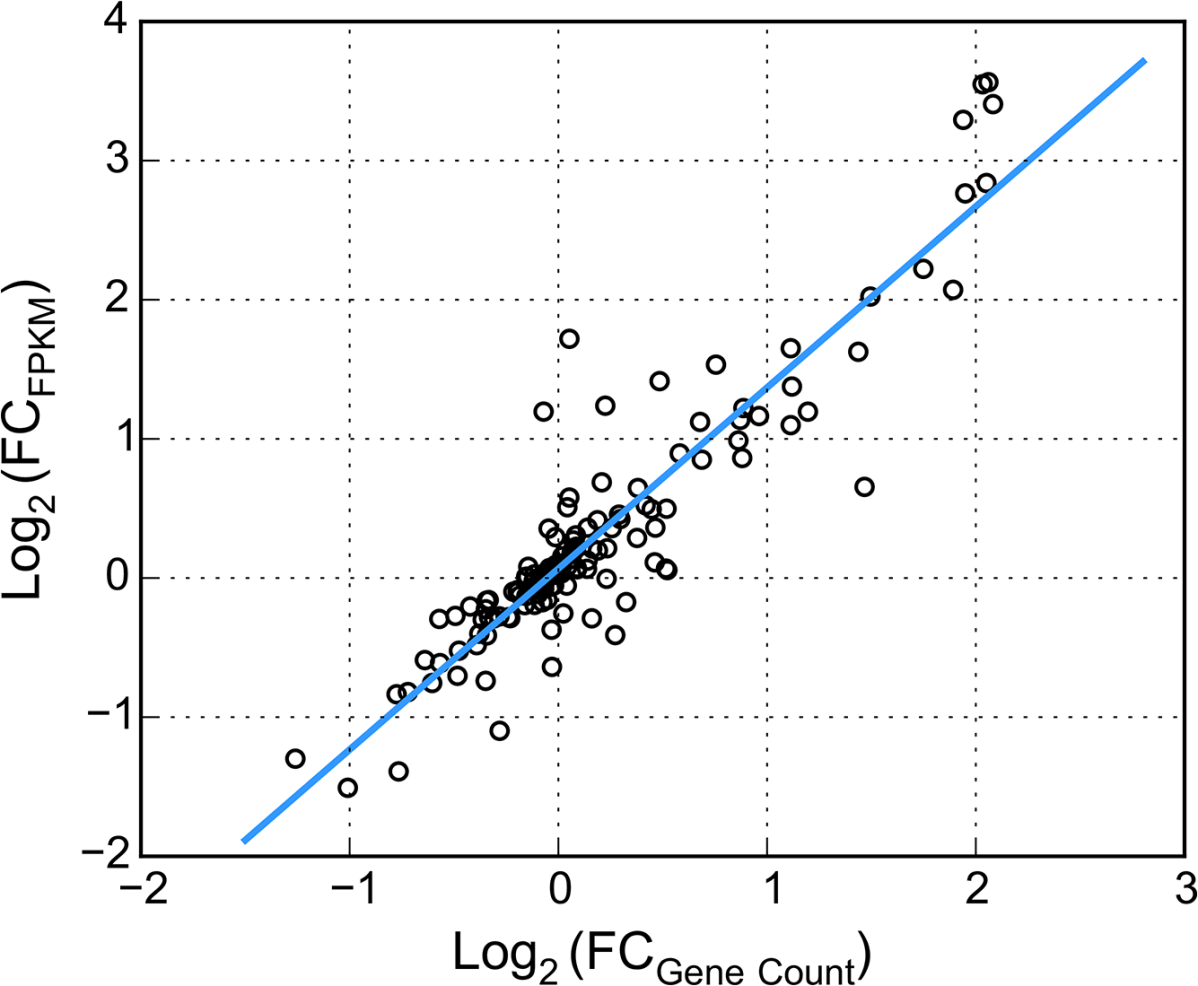
Correlations of log2 fold-change between RNA-seq FPKM and Nanostring gene count.

We confirmed the gene expression results obtained from RNA-seq data using the Nanostring nCounter analysis system. The normalized Nanostring gene count showed a strong correlation with the FPMK values of RNA-seq. The correlation coefficient between fold change of the gene count and fold change of FPKM values was 0.92 (Fig 3). Based on the 31 DE genes from RNA-Seq analysis, the correlation between FPMK and gene count was 0.93 (S2 Fig). The results showed a high consistency between the two technologies, and confirmed that the gene expression data of RNA-Seq was reliable.

## Discussion

Consistent with previous observations [26–28], our data showed a negative correlation between fatness and FE. Comparing the HFE and LFE groups, the BWs were not significantly different, but FC, WG and abdominal fat percentage were significantly different. The LFE chickens had more feed intake (1.15 fold) and deposited less breast muscle (0.93 fold) but more abdominal fat (1.55 fold). The LFE chickens appeared to partition the energy obtained from diet to accumulate more fat but build less breast muscle than HFE chickens. RNA-seq analysis of the selected chickens revealed that genes involved in *de novo* triglyceride synthesis, cholesterol synthesis, lipid transport, and lipid stabilization were up-regulated, whereas genes involved in lipid hydrolysis and lipid reverse efflux were down-regulated in the abdominal adipose tissue of LFE birds (Table 6). Also, several genes related to coagulation, immune system, amino acid metabolism, and carbohydrate metabolism were differentially expressed between LFE and HFE groups.

**Table 6:** Summary of DE genes involved in lipid accumulation.

Functional category	Gene Name	Full Name	RNA-Seq fold change
Fatty acid transportation	*FABP1*	*fatty acid binding protein 1*	**↑**5.7
Stabilization of fatty acid	*ALB*	*albumin*	**↑**9.8
*de novo* triglyceride synthesis	*SCD*	*stearoyl-CoA desaturase*	**↑**2.6
	*AGPAT9*	*1*-acylglycerol-*3-phosphate O-acyltransferase 9*	**↑**1.4
	*DGAT2*	*diacylglycerol O-acyltransferase homolog 2*	**↑**3.1
Triglyceride hydrolysis	*MGLL*	*monoglyceride lipase*	**↓**1.4
Cholesterol synthesis	*DHCR24*	*24-dehydrocholesterol reductase*	**↑**1.7
	*HSD17B7*	*17-beta hydroxysteroid (17-beta) dehydrogenase 7*	**↑**1.7
	*CYP51A1*	*cytochrome P450*, *family 51*, *subfamily A*, *polypeptide 1*	**↑**1.5
	*HMGCS2*	*3-hydroxy-3-methylglutaryl-CoA synthase 2*	**↑**2.4
Cholesterol transport	*APOA1*	*apolipoprotein A-I*	**↓**1.6
	*LCAT*	*lecithin-cholesterol acyltransferase*	**↓**1.7
Steroidogenesis	*CYP11A1*	*cytochrome P450*, *family 11*, *subfamily A*	**↓**2.0
	*TSPO2*	*translocator protein 2*	**↓**2.2
Adipogenesis	*PPARG*	*peroxisome proliferator-activated receptor gamma*	**↑**1.6
	*FSTL1*	*follistatin-like 1*	**↓**1.5
	*KLF15*	*kruppel-like transcription factors 15*	**↑**1.5

**↑** indicates up-regulation in LFE group, fold change = FPKM_LFE_ / FPKM_HFE_

**↓** indicates down-regulation in LFE group, fold change = FPKM_HFE_ / FPKM_LFE_

### Triglyceride and cholesterol metabolism

By comparing the gene expression in the adipose tissue of the HFE and LFE groups, we identified the DE genes that may be responsible for the differences in fatness. IPA predicted the accumulation of lipid in LFE group is activated (activation z-score: 2.14). (Fig 2A). *SCD*, *AGPAT9*, and *DGAT2* are three important genes involved in *de novo* triglyceride synthesis. All of the three genes were expressed at higher levels in LFE birds, with a fold change (FPKM_LFE_ / FPKM_HFE_) of 2.6, 1.4 and 3.1, respectively. SCD is a lipogenic enzyme located on the membrane of the endoplasmic reticulum (ER). It catalyzes the rate-limiting step of mono-unsaturated fatty acid (MUFA) biosynthesis from saturated fatty acids (SAFAs) [29]. The expression of *SCD* is closely associated with adiposity in previous studies [15,30]. AGPAT9 catalyzes the first, and DGAT2 catalyzes the last step of triglyceride synthesis. DGAT2 is located in the proximity of SCD in the ER membrane, where SCD facilitates substrates transport for triglyceride synthesis [31]. *DGAT2* expression could be affected by available energy sources in cells. In fasted chickens, the expression levels of *DGAT2* in adipose tissue were much lower [16]. According to our FC records, the LFE group consumed 1.15 fold (i.e., ~430 grams) more feed than did HFE group. The relatively more abundant dietary energy resource might promote the *de novo* biosynthesis of triglycerides in the adipocytes of LFE birds through up-regulation of *DGAT2*. Consistent with our results, *SCD* and *DGAT2* were found down-regulated in Leghorn, a relatively lean line, when compared with a relatively fat line, i.e. a commercial broiler line [18].

FABP1 functions as a carrier protein for fatty acids, which transfer the fatty acids across the cell membranes. Increased *FABP1* expression was found in the adipose tissue of obese people who had high acylation stimulating protein and high triglyceride levels in a fasting plasma test [32]. More FABP1 might facilitate the transfer of fatty acid uptake in the adipose tissue and contribute to the accumulation of triglycerides. On the other hand, the expression levels of *ALB* were higher in LFE group. Knockdown or point mutations of the fatty acid binding site of albumin in cultured adipocytes suppressed lipid droplet formation, suggesting the role of albumin is to promote the formation of lipid droplets by binding to fatty acids [33]. The higher expression of albumin in LFE group suggests a similar lipid-stabilizing role of albumin in adipocytes of chickens, as exists in mammals.

Several genes involved in cholesterol metabolism were differentially expressed between HFE and LFE group. Adipose tissue is the largest site for free, un-esterified cholesterol storage [34]. There were several cholesterol biosynthesis pathways over-represented in LFE group, and all four DE genes involved in those pathways were up-regulated, suggesting a relatively higher cholesterol synthesis activity in the adipocytes of LFE group (Table 6). In particular, the expression of *3-hydroxy-3-methylglutaryl-CoA synthase 2 (HMGCS2*) in LFE group was 2.4 fold (FPKM_LFE_ / FPKM_HFE_) higher than that of HFE birds. HMGCS2 catalyzes the production of 3-hydroxy-3-methylglutaryl-CoA (HMG-CoA), a precursor for the rate-limiting step of cholesterol biosynthesis. The expression of *24-Dehydrocholesterol Reductase* (*DHCR24*), which encodes for the final enzyme in the cholesterol biosynthesis pathway, was 1.7 times higher in LFE group. Furthermore, we found several down-regulated genes that may contribute to cholesterol deposition through lower conversion in LFE chickens (Table 6). As a major component of HDL, APOA1 starts the formation of HDL by lipidation, and LCAT is responsible for turning the lipidated particles into spherical shapes [35]. Previous studies have shown that *APOA1* expression in liver was higher in a fat line of chickens [36,37]. Down-regulation of the expression of *APOA1* and *LCAT* may affect the formation of HDL, which reduces the capacity of reverse transportation of cholesterol from adipose tissue to liver and muscle, and results in more free cholesterol stored in the abdominal fat of LFE birds. As an endocrine organ, a very important function of adipose tissue is the production of steroid hormones. We found the expression of *CYP11A1* was lower in LFE group. The enzyme encoded by *CYP11A1*, P450scc, is the rate-limiting enzyme for converting cholesterol to pregnenolone (3β-hydroxypregn-5-en-20-one) [38]. Pregnenolone is a neurosteroid and a precursor of several steroid hormones. With a fold change (FPKM_HFE_ / FPKM_LFE_) of 2.0, decreased expression of *CYP11A1* in LFE chickens may reduce the rate of cholesterol conversion to pregnenolone and cause more cholesterol to be stored in the adipocytes in LFE group. Collectively, our data indicates that more triglycerides and cholesterol were stored in the form of lipid droplets, causing hypertrophic growth of adipocytes.

### Upstream regulators of cholesterol synthesis pathway

Sterol regulatory element-binding proteins (SREBPs) and INSIGs are key transcription factors in the regulation of cholesterol metabolism. IPA predicted SREBP1 (z-score = 2.529, overlap p-value = 3.43E-04) and SREBP2 (z-score = 2.449, overlap p-value = 5.23E-05) as being activated but INSIG1 as being inhibited in the abdominal fat tissue of LFE birds **(** Fig 2B **)**. In mammals, the SREBP1 and SREBP2 genes encode for three different protein isoforms with different target genes [39,40]. Located on the ER membrane, INSIG1 regulates cholesterol biosynthesis by sensing the sterol level. With sterols present, INSIG1 binds to the complex of SREBP and SREBP chaperone (SCAP) and keeps it on the ER membrane. Without sterol, INSIG1 is isolated and thus is subjected to ubiquitination and degradation [41]. The free SERBP migrates to Golgi to be further processed and, subsequently, enters the nucleus and activates genes involved in cholesterol and fatty acid metabolism [42,43], including *INSIG1* gene. In turn, *INSIG1* expression reduces lipid production and adipogenesis *in vitro* [44]. As a negative regulator of cholesterol synthesis, inhibition of INSIG1 by degradation may trigger activation of SREBP1 and SREBP2, and assist in the higher accumulation of cholesterol. In turn, SREBP proteins activate the expression of *INSIG1* to compensate for the degraded INSIG1 and maintain the level of INSIG1 [41]. Consistent with that, *INSIG1* was up-regulated in LFE chickens (FPKM_LFE_/FPKM_HFE_ = 1.4). Hence, a self-regulating loop may be present in adipocytes to maintain the cholesterol amount in an appropriate level.

### Hyperplastic growth

Adipocyte hypertrophy might be a prominent contributor to abdominal fat mass [45], but adipocyte hyperplasia could also play a role. In particular, the adipose tissue of broiler chickens have hypertrophic and hyperplasic growth until 14 weeks of age [46]. In the present study, the LFE group had a higher expression level of *PPARG* (FPKM_LFE_ / FPKM_HFE_ = 1.56 fold). PPARG is an extremely important regulator in lipid metabolism and adipogenesis. It is required for the development of adipose tissue [47], as it is involved in both differentiation of preadipocytes and proliferation of adipocytes. Previous research has shown that *PPARG* expression in the adipose tissue of chickens is strongly correlated with abdominal fat pad weight [48]. It’s possible that higher expression of *PPARG* increases the differentiation and proliferation of adipocytes, causing a multiplication of adipocytes in LFE birds. In agreement, *follistatin-like 1* (*FSTL1*) was expressed lower in LFE group. The expression of *FSTL1* is down-regulated during pre-adipocyte to adipocyte differentiation [49]. Additional support of hyperplasic growth comes from up-regulation of *Kruppel-like transcription factors 15* (*KLF15*). KLF15 is recognized as a regulator of PPARG, reflected by the strong correlation between *KLF15* and *PPARG* expression in our data (r = 0.80). *KLF15* expression is up-regulated during preadipocyte differentiation, and interruption of KLF15 decreases *PPARG* expression and affects differentiation [50]. Based on our collective data, we propose that hyperplasia may also contribute to the higher accumulation of abdominal fat mass in LFE birds.

### Amino acid and carbohydrate metabolisms

A few DE genes encodes for key enzymes in amino acid and carbohydrate metabolism. Three genes [*tyrosine aminotransferase* (*TAT*), *phosphoserine phosphatase* (*PSPH*), and *argininosuccinate lyase* (*ASL2*)] were associated with the biosynthesis of tyrosine, serine, and arginine, respectively. Two DE genes were found involved in carbohydrate metabolism. *Amylase alpha 2A* (*AMY2A*) was expressed 2.2-fold (FPKM_LFE_ / FPKM_HFE_) higher in LFE chickens. AMY2A catalyzes the first step in the breakdown of large polysaccharides, including glycogen. The restoration of lipid for lipid-depleted adipocytes requires the accumulation of a certain amount of glycogen, possibly followed by glucose-to-lipid conversion [51]. The higher expression of *AMY2A* possibly indicates that glucose-to-lipid conversion is more active in LFE chickens. In addition, the expression of *AHSG* was found with great difference between HFE and LFE group (FPKM_LFE_ / FPKM_HFE_ = 10.4). Encoded by *AHSG*, alpha-2-HS-glycoprotein is involved in glucose metabolism and the regulation of insulin signaling. Knockout of *AHSG* induces glucose tolerance and decreased body fat [52]. AHSG may affect glucose uptake and lipid oxidation in adipocytes through regulation of adiponectin and may have an impact on fat deposition in LFE chickens.

## Conclusion

In summary, our FE tests of commercial broiler chickens suggest a moderate correlation between abdominal fat percentage and feed efficiency. Compared with HFE chickens, LFE chickens had higher feed intake and deposited more abdominal fat but less breast muscle. The higher feed intake may play a role by increasing the lipid concentration in blood circulation and promote fat deposition in LFE birds, but other triggers of differential gene expression between HFE and LFE chickens remain to be studied. To the best of our knowledge, this is the first study of the relationships between gene expression in adipose tissue and FE. The results of our study provide mechanistic insights into the biological basis of differences in adiposity between HFE and LFE chickens. In addition, as the adipose tissue of human and chicken share certain physiological features and gene homology, our findings regarding chicken adipose tissue could potentially be useful for studies of obesity in humans.

## Supporting Information

S1 Fig The total number of sequence reads for each sample. (TIF)

S2 Fig Correlations of log2 fold change (FC) of DE genes between RNA-seq FPKM and Nanostring gene count. (TIF)

S1 Table The detailed phenotypes of the chickens selected for RNA-Seq. (XLSX)

S2 Table NanoString genes sets and gene counts for each sample. (XLSX)

S3 Table Correlation coefficients between WG, BW, FC, Fat%, RFC and FCR. (XLSX)

S4 Table Correlation coefficients of gene expression profiles among LFE chickens. (XLSX)

S5 Table Full list of DE genes and the average FPKM of each group. (XLSX)
